# Effectiveness of silica based sol-gel microencapsulation method for odorants and flavors leading to sustainable environment

**DOI:** 10.3389/fchem.2015.00042

**Published:** 2015-08-11

**Authors:** Muhammad Aqeel Ashraf, Aysha Masood Khan, Mushtaq Ahmad, Maliha Sarfraz

**Affiliations:** ^1^Department of Geology, Faculty of Science, University of MalayaKuala Lumpur, Malaysia; ^2^The Centre for Research in Biotechnology for Agriculture, University of MalayaKuala Lumpur, Malaysia; ^3^The Centre for Research in Waste Management, University of MalayaKuala Lumpur, Malaysia; ^4^Faculty of Science and Natural Resources, Universiti Malaysia SabahKota Kinabalu, Malaysia; ^5^Department of Plant Sciences, Quaid-i-Azam UniversityIslamabad, Pakistan; ^6^Institute of Pharmacy, Physiology and Pharmacology, University of AgricultureFaisalabad, Pakistan

**Keywords:** microencapsulation, sol-gel, stabilization, odorant, environmental benefits

## Abstract

Microencapsulation has become a hot topic in chemical research. Technology mainly used for control release and protection purposes. The sol-gel micro encapsulation approach for fragrance and aroma in porous silica-based materials leads to sustainable odorant and flavored materials with novel and unique beneficial properties. Sol-gel encapsulation of silica based micro particles considered economically cheap as capital investment in manufacturing is very low and environmentally friendly. Amorphous sol-gel SiO_2_ is non-toxic and safe, whereas the sol-gel entrapment of delicate chemicals in its inner pores results in pronounced chemical and physical stabilization of the entrapped active agents, thereby broadening the practical utilization of chemically unstable essential oils (EOs). Reviewing progress in the fabrication of diverse odorant and flavored sol-gels, shows us how different synthetic strategies are appropriate for practical application with important health and environmental benefits.

## Introduction

Encapsulation technology arouse as a sparkle in recent world accounting significant health and environmental benefits. Industry grow with wide range of applications in all fields specifically, Deodorants, food, oils, synthetic nitro and polycyclic musk's, detergents, cosmetics (perfumes), personal care (hand and body wash, toothpaste, etc.), food (flavors), and home care (laundry and detergents) etc. (van Soest, 2007). Earliest work reported in literature on microencapsulation dates back to 1980s. Encapsulation defined as entrapment of one substance (active agent) into another substance. Industry involves materials such as organic polymers, biopolymers, silica, degradable, and non-degradable. Different encapsulation technologies introduced during last decades. Of these sol-gels microencapsulation proves promising to stabilize natural fragrances and aromas for a longer time (Ciriminna and Pagliaro, 2013). Technology introduced about 60 years ago in the field of biotechnology to make production processes efficient and stable during whole operation and at the time of final product formation (Tabassum et al., 2014).

### Silica based sol-gel microencapsulation

Microencapsulation has become a hot topic in chemical research. Technology mainly used for control release and protection purposes. According to Gosh, microencapsules related to enclosure of micrometer size solid, liquid or gas in inert shell, in turns protect and isolate from external environment (Ghosh, 2006). A convenient and alternative method to ordinary microencapsulation for flavors and fragrances is sol-gel silica based microencapsulation taking place at room temperature, preventing degradation of compounds, applied for the first time in 1987 ranging from irregular silica xerogel particles to sophisticated core shell particles resulting in commercialization of sol-gel encapsulated odorants and flavors leading to fragrances and aromas. Fragrances and flavors, aromas and odorants comprise mainly conjugated double bonds which are easily oxidized. Sol-gel encapsulation normally carried out with emulsion consisting of two immiscible phases and prepared in the presence of surface active ingredients. Ionic and non-ionic surfactants widely used in making silica based micro particles (Nouria et al., 2012).

**Figure 1:**
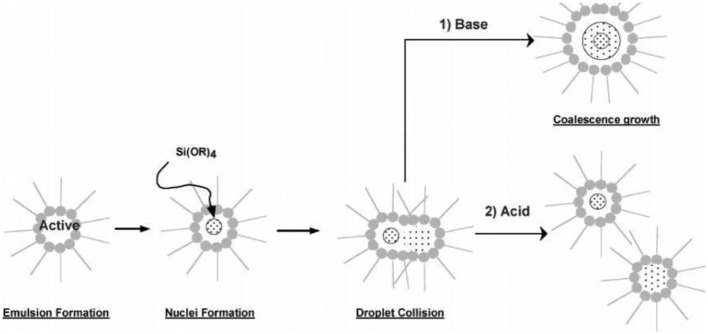
**Proposed mechanism for particle formation under acidic and basic conditions (Finnie et al., 2007)**.

Microencapsulation technique divided into two basic groups' i.e., physical and chemical having number of applications. Important chemical microencapsulation technology consists of *in situ* processes such as emulsion, suspension, precipitation, dispersion polymers, and interfacial polycondensation while physical properties involve methodologies such as spray drying and spinning disk. In 2004, Liposome entrapment and spinning disk were dominant technologies but now sol-gel microencapsulation is an emerging technology. Emulsion droplets provide micro reactor environment for condensation and hydrolysis of Si alkoxide. Silica micro particles even in corrosive environment show enhanced chemical stability. By changing pH of sol-gel materials, silica particles with wide control of size and shape can be obtained. Technique built an oxide cage around polar droplet, yielding particles with sizes same as droplets so by changing solvent surfactant combination different particle sizes can be obtained. Fast dissolution and condensation achieved by using base catalysis in sol-gel encapsulation leading to inhomogeneous system with dense silica particles during collision of droplets and by the ripening shown in Figure 1. Generally, it is concluded that base catalyzed silica particles in water/organic (W/O) micro emulsions have low or negligible porosity but to obtain porous particles two step sol-gel polycondensation process used which involve acidic hydrolysis followed by base (Ullah et al., 2013).

**Figure 2:**
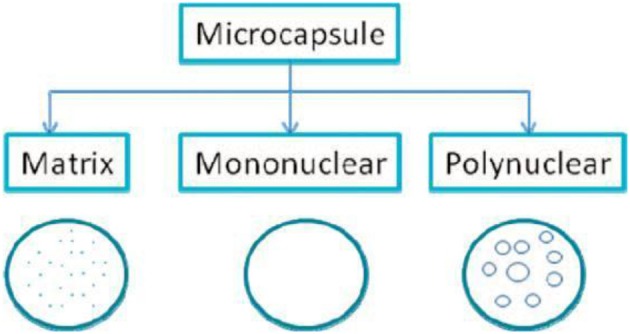
**Morphology of microcapsules (Ciriminna and Pagliaro, 2013)**.

Based on core material and deposition process of shells, morphologically microencapsules classified as matrix, mononuclear, and polynuclear as shown in Figure 2. It controls the function and utility of sol-gel materials on nano, meso, and nano scale. It seems one of the promising encapsulation technique compared to polymers in the presence of organics in nano-structured porous silica possessing high transparency, non-toxicity, low chemical interaction, thermal protection, biocompatibility resistant to oxidation, high mechanical strength, ineffectiveness to pH and less variations with temperature (Parveen et al., 2015). Other characteristics include water bodies and oil based capacity with less micro bacterial attack. A big deal of this method relates to the stabilization and entrapment of actively released compounds, an important and efficient purpose of this industry. In industrial sector, sol-gel microencapsulation becomes most relevant chemical technology with numerous applications.

### Historical background

Concept of incorporation of organic chemistry to inorganic glass (amorphous silica) given by Avnir and co-workers in 1984. Simple addition or entrapment of organic molecules in inner porosity of silica used as matrix at the onset of sol-gel process can be shown as below in Equation (1) (Avnir et al., 2001).

(1) $$\begin{array}{l} \text{Si}{\left({\text{OR}}^{\prime }\right)}_{4}+\text{RSi}\left({\text{OR}}^{\prime }3\right)+\text{H2O}\underset{\text{Cosolvent}}{\to }\text{dopant}@\\ \;\left[{\text{RSiO}}_{n}{\text{H}}_{m}\left({\text{OR}}^{\prime }\right)q\right]p \end{array}$$

**Figure 3:**
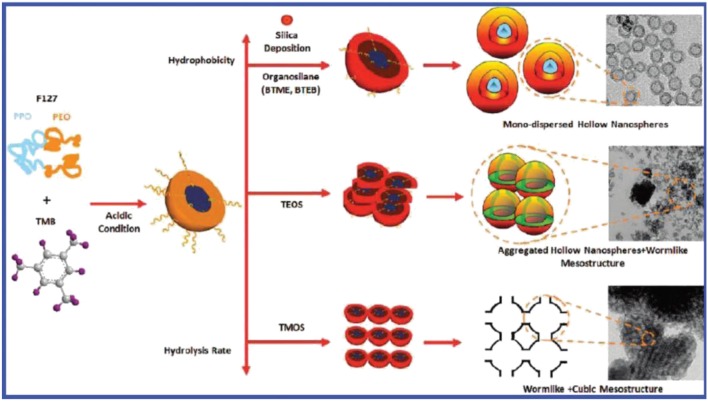
**Formation mechanism of ORMOSIL nanoparticles and the relation between the morphology and the silane precursors**.

Above equation represent broad range of ORMOSIL preparations. Silica cage provide both physical and chemical stability to dopant molecules. In 1987 Japanese research company reported for the first time sol-gel entrapment of fragrances and aromas in silica xerogel particles while in 1992, Reineccius reported limonene encapsulation on silica with enhanced resistance to oxidation than to carrier over organic food (Bolton and Reineccius, 1992). Organically modified silicates (ORMOSIL) matrices encapsulated with geranil, menthol and β-ionone in 1997 by Carturan and his co-workers. Results show slow release of actives and more stability. Formation of ORMOSIL nano particles is shown in Figure 3.

Kinetic study uniquely attributes differences in chemical interaction of organic molecules with matrix and its porosity. Organic liquids encapsulated in silica based xerogels and thin film coatings from hydrolyzed silica sols studied. Several factors play their role in the retention of fragrances and aromas as it is considered a complex phenomenon. Concept of entrapment in microregions seems to prevail for sol-gel silicates. Hydrolysis ratio and blank powder porosity influence aroma retention. Decreased porosity leads to enhanced nanoconfinement resulting in increased retention capacity by capillary force and decreased diffusional loss. Encapsulation of different chemicals such as esters, alcohols, aldehydes, terpenes etc., *in silica* for aroma generation is brought by tetraethyl orthosilicate hydrolysis (Veith and Paratisinis, 2004). Through sol-gel technology, parameters such as porosity, pore-size distribution, particle morphology can be conveniently optimized and controlled. Aroma load found in direct relation with retention capacity with maximum retention shown by denser silica matrices. In food and pharmaceutical industries porous sol-gel particles used as encapsulated matrices. Generally, Flavors composed of many chemical compounds responsible for fragrance and aroma, entrapment of other species change the retention capacity affecting aroma. Initial mass fraction for different components can be determined by Equation (2).

(2) $$x_{i0}=\frac{m_{i0}}{\sum_{I}m_{i0}+m_{S0}}$$

Where *m*_*i*0_ = input mass of each aroma component, *m*_*S*0_ =the dry amount of silica, While total initial aroma mass fraction defined as Equation (3)

(3) $$x_{tot}=\frac{\sum_{i}m_{i0}}{\sum_{i}m_{i0}+m_{S0}}$$

Extraction and GC analysis performed for aroma load show total mass of sol-gel powders best described by following Equation (4)

(4) $$m_{\text{E}}=m_{\text{SE}}+m_{\text{W}}+\sum_{i}mi$$

*m*_*i*_ = mass of the aroma component in the extraction sample, *m*_*W*_ the mass of water, *m*_*SE*_ the mass of dry silica subjected to extraction Aroma retention of different components i in matrix found by Equation (5)

(5) $$R_{i}=\frac{m_{i}/m_{\text{SE}}}{m_{i0}/m_{\text{S0}}}$$

Where the subscript 0 stands for the initial conditions (Veith and Paratisinis, 2004).

### Effect of particles of different chemical species on aroma retention *in silica*

#### Esters

Encapsulation of esters in sol-gel made silica done by using ethylhexanoate, ethyl butanoate, and ethyl octanoate. From the results it is shown that average retention capacity increase with decrease in polarity. Bigger molecules with less volatility retained for much longer time. Smaller molecules in densest sample recovered to smaller extent.

#### Aldehydes

**Figure 4:**
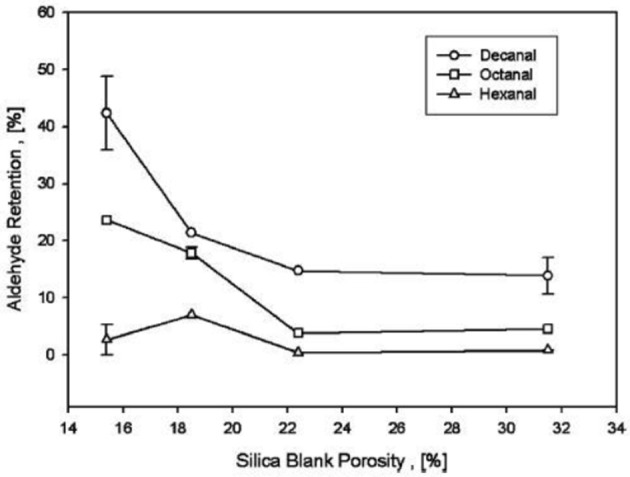
**Shows individual aldehyde retention in silica as a function of blank porosity with initial load of 20 w%**.

Mixture of aldehydes with initial loading of 5, 10, and 20 w% encapsulated in sol-gel made silica particles. Average retention of each component is shown by the Figure 4 indicate that decreased blank porosity cause an increase in retention time. With initial load of 20 w% in densest sample maximum aldehyde load of 5 w% obtained. Aldehydes can form hemiacetals as well.

#### Alcohols

**Figure 5:**
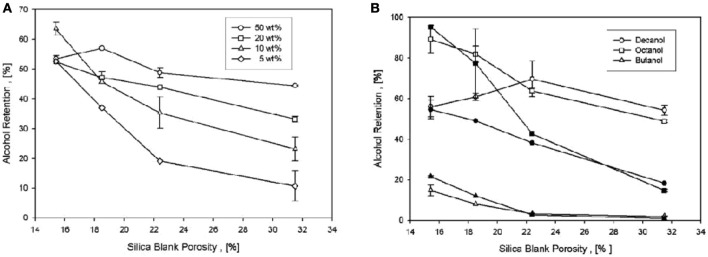
**(A)** Average alcohol retention in silica as a function of the blank porosity for total initial loads of 5, 10, 20, and 50 wt%. **(B)** Individual alcohol retention in silica for a total initial load of 20 wt% (open symbols) and 5 wt% (filled symbols) (Veith and Paratisinis, 2004).

Alcohol retained better than other compounds and chemically reacts with silica (Hair, 1967). For alcohols encapsulation in silica, mixture of butanol, octanol, and decanol preferred with total initial load of 5–50 w%. Results shown by Figure 1 describe decrease retention with increased porosity however increase with initial load in contrast to carbohydrate matrices which shows inverse relation with load (Reineccius and Coulter, 1969). If porosity lessened to one half for an initial load of 5 w% retention capacity enhanced about five folds. Retention capacity as a function of blank silica porosity shown by Figure 5 indicates better retention of bigger molecules like octanol and decanol than smaller molecules like butanol. Overall, it is suggested that enhanced alcohol retention obtained by decreasing particle pore size and increasing aroma load.

Two hydroxyl groups of alcohols and silica react to form stable bond with the release of water molecule. Bonding on silica surface leads to physicosorption (Boutboul et al., 2002). Aldehydes and alcohols containing carbonyl and carboxyl group show similar interaction with silica. Generally, retention seems linear with molecular weight for esters, Aldehydes, and alcohols due to Van der Waals forces indicating physical adsorption. Concept of encapsulation refer to adsorption of chemical species on silica surface, forming associations turning carrier to much more stable state in confined pore space.

### Formation of hollow spheres from W/O emulsions

**Figure 6:**
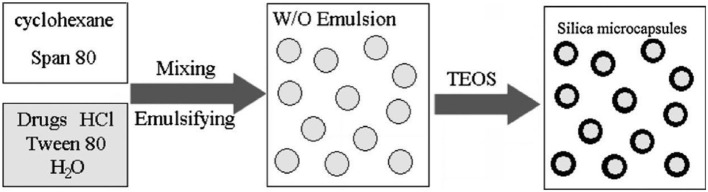
**Schematic procedure for direct encapsulation of water-soluble drugs into silica microcapsules**.

High dopant storage capacity and excellent sustained released properties obtained by hollow silica spheres are ideal for medical and drug application. Using water phase in W/O emulsion as a container prior to addition of TEOS direct encapsulation and storage of water soluble molecules can be achieved then Si-alkoxide in oil phase introduced and diffused through surfactant wall in water droplet forming gelatin with stable microcapsules at the interface can be shown in Figure 6.

### Interaction of odorant molecules with silica sol-gel cage

**Figure 7:**
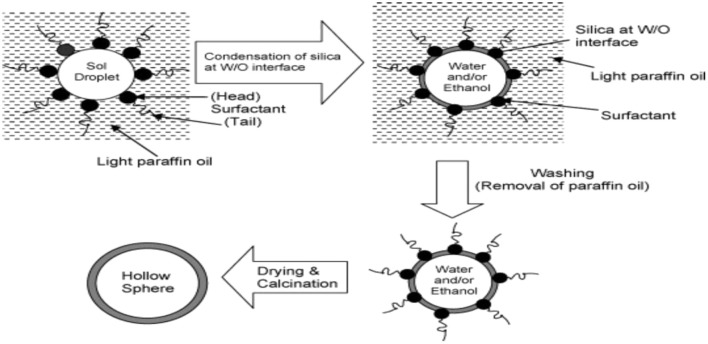
**Schematic representation of the formation of hollow silica spheres**.

Sol-gel processing of TEOS in silica entrapping different odorant molecule involves chemical reaction between dopant and surface Si-OH groups. Alcohol show highest retention capacity and stay than aldehydes, esters, and terpenes due to the formation of hydrogen bonds with Si-OH group through its oxygen free π- electrons. Porosity affects retention time that increased with decreasing porosity. Release kinetics affected by initial load where dopant entrapped molecules affect the six of sol-gel cage, acting as tempelate. Research concludes large cage cause quick aroma release than smaller one. Micro emulsions used instead of water and alcohol for sol-gel polycondensation these days with effective protection and separation of dopant molecules (Pagliaro et al., 2011). Generally core shell dissolution or mechanical rupture as having advantage of difference in surface morphology by emulsification or casting with poor volume and reduced surface area. Australian Geramisphere presented liquid limonene encapsulated silica with striking microtomography image shown in Figure 7.

**Scheme 1:**
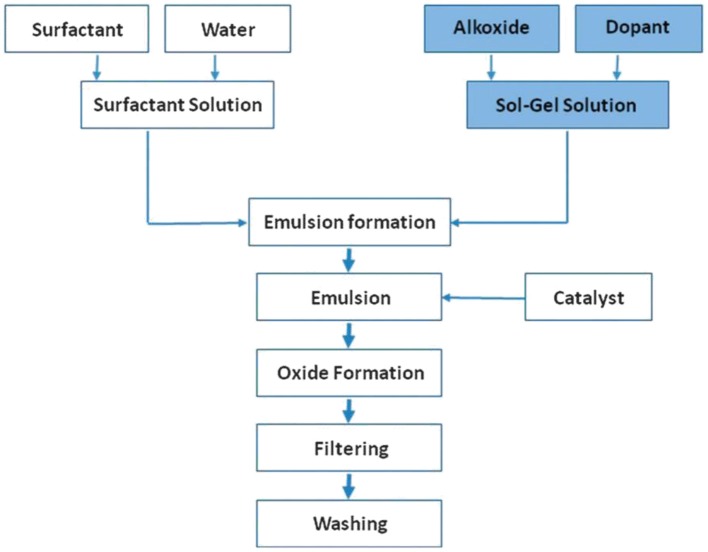
**Preparation of doped core/shell particles via the sol-gel process.** Key is the combination of: emulsion-polymerization and sol-gel chemistry. The RT production of glass now takes place in the emulsion droplets acting as micro-reactors, affording microencapsulated actives of all sort (Ciriminna et al., 2011).

Sol-gel microencapsulation demonstrated first by Takahashi and co-workers in 1998, further extended and applied on encapsulation of flavors, oils, protein, vitamins, biomolecules etc., demonstrating the formation of homogeneous micro particles using high water to surfactant ratio. Avnir and co-workers extended their work to molecular encapsulation describing the scope of diverse application of O/W emulsion for doping as shown in Scheme 1 below:

Doped silica microspheres can be produced from surfactants by mixing TEOS with aqueous HCl in acid catalyzed sol added drug using two step sol-gel process followed by base and further processes. Large size microspheres widely different from xerogel granules obtained as shown in Figure 10.

Size normally depends on the speed at which emulsification done. With increase in speed, size of emulsification decreases. ORMOSIL micro particles mediated by surfactants were synthesized by Henbrite and co-workers.

### Control release of sol-gel encapsulated particles

Release rate in full or hollow microsphere independently from particle size can be controlled by diffusion and is enhanced compared to leaching from xerogel particulate materials, estimated by sol-gel chemistry and internal microstructure. Generally released rate reduced by hydrophobicity of silica matrix (Fanun, 2008). Baker Lonsdal model for spherical shape diffusion matrices shown by Equation (6).

(6) $$3/2\left[1-{\left(1-{\text{M}}_{1}/{\text{M}}_{0}\right)}^{2/3}\right]-\text{M1/M0 }=\text{ kt}$$

Hollow mesoporous silica possesses high storage capacity. Calcination in the formation of uniform hollow sphere from W/O emulsion during sol-gel oxide formation prevents doping of direct microcapsules. Shi and co-workers demonstrated ways for controlled release polyelectrolyte multilayer coating by addition to suspension making capsule to act as switch for drug release (Zhu and Li, 2005).

### Applications of sol-gel encapsulated particles

Porous sol-gel particles having high strength and thermal stability, hydrophilic nature, bio-compatibility, rapid biodegradability, fast dissolution in butter at pH 7.4 can be easily suspended in water, extreme protection of dopant from acids, base, detergents, and enzymatic degradation best applied in health care, cosmetics, food, and special chemicals (Finnie et al., 2009).

#### Silica based sol-gel microencapsulation in perfumes, food, and cosmetics

**Figure 8:**
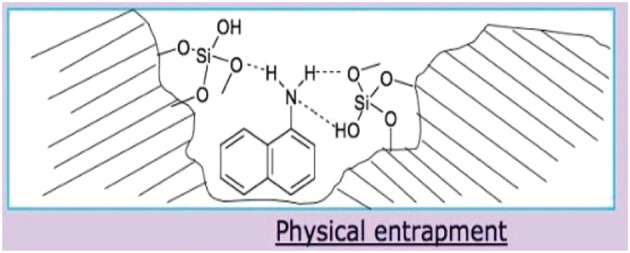
**Physical entrapment of a dopant molecule in the inner porosity of a sol-gel silica matrix [Image courtesy of Prof. D. Avnir]**.

***Application of sol-gel microcapsules in perfumes:*** Microencapsulation technology also successfully applied on fragrance mixtures such as terpenes with essential oils (EOs) and bergamot oil in different silica based microcapsules widely applied in perfumes (Dugo and Bonaccorsi, 2013). EOs are ancient and known class of compounds, natural products of plants with multi component system, divided into two major groups i.e., hydrocarbons and oxygenated compounds with many active phytochemicals (Porto et al., 2009). Distinguished features include antimicrobial, antisepticidal, herbicidal, repellant, antioxidant and as alternative of materials in agriculture, industries, cosmetics, medicine, food, and pharmaceuticals. These disciplines involve sol-gel microencapsulation technique to convert liquid form to solid and controlled release of bioactive molecules. Most of the EOs show chemical instability toward light, temperature, air, and moisture but put great impact in many essential oil domains. Lavender and mint, two EOs become more protected and resistant toward environmental conditions using sol-gel microencapsulation technology involving cyclodextrin formulating original process in silica matrices. B-cyclodextrin also possesses affinity to form inclusion complexes(IC) with EOs proved by chromatographic and spectroscopic techniques (Figure 8).

**Figure 9:**
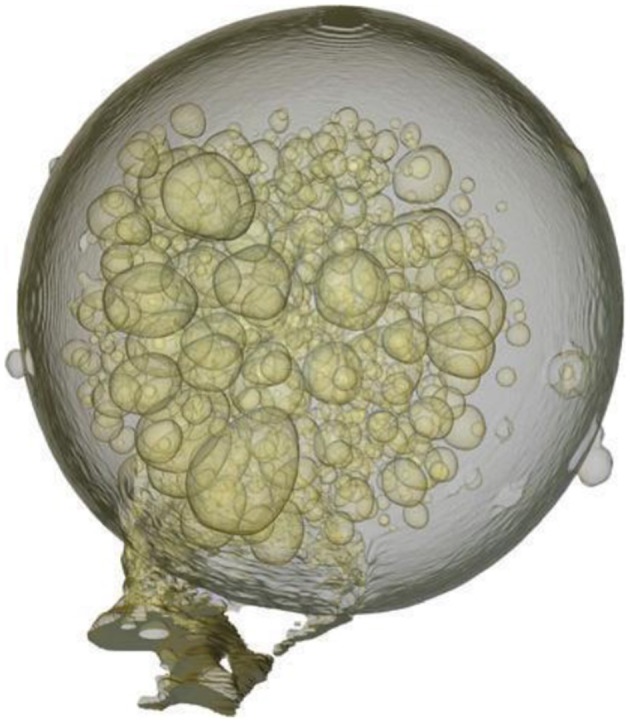
**X-ray micro-tomography image of a Ceramisphere (a trade mark of Ceramisphere Pty Ltd) containing encapsulated limonene clearly seen as liquid bubbles**. (Ciriminna and Pagliaro, 2013) Scheme also describes the general mechanism of encapsulation in glass shown as below.

Lot of study done on polyurea and solid-lipid nanoparticles microencapsulation however molecular encapsulation by cyclodextrin present modification of physico-chemical properties of EOs (Lai et al., 2006; Arana-Sánchez et al., 2010; Ahmad et al., 2014). Sol-gel microencapsulation provide advantage of entrapping of molecules by forming inclusion complexes between EOs as guest molecules and β-CD as host in porous silica, turning to more stable and making resistant toward environmental conditions. Silica acts as protector for EOs. Novelty in this technology for EOs based on chromatographic analysis using water as a solvent (Rǎileanu et al., 2013). Monoterpene rich EOs used as flavor ingredients having greater polarity show more solubility in aqueous medium possess high rate of diffusion results in greater loss however compound retention is a function core molar volume. Sumac flavor encapsulation in sodium chloride successfully done but due to salty and acidic nature salted cookies, crackers, salads etc., cannot be processed (Gharsallaoui et al., 2007). Recently lots of applications occur for silica based microencapsulation showing the advancement of this technique for perfumes, aromas, and fragrances (Figure 9). Giraudan, Flavor and Fragrance Company worked on the enhancement of mechanical strength toward sol-gel capsules. Gelatin core shell capsules entrap oil active ingredients by coacervation, making hydrogel shells mixed with TEOS turning it to silica based composite shells releasing small amount of ingredients making it beneficial for food (Mateen et al., 2015).

**Figure 10:**
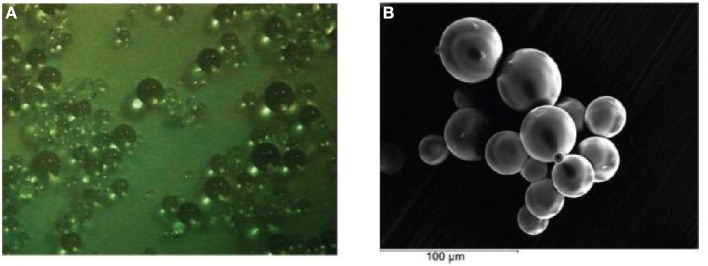
**Optical and SEM micrographs of emulsified acid-base catalyzed silica microspheres**. The optical (original magnification: 60 ×) and SEM (original magnification 600 ×) images. **(A)** Show the appearance of microspheres in the size range of 10–40 μm in diameter. **(B)** Show the appearance of microspheres in the size range of 100–300 μm in diameter.

#### Application of sol-gel particles in food

**Figure 11:**
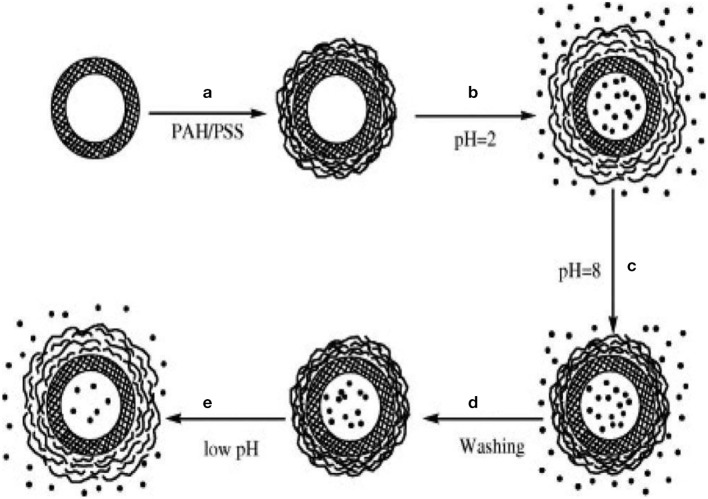
**Polyelectrolyte multilayers (PEM), consisting of sodium polystyrene sulfonate (PSS) and poly(allylamine hydrochloride) (PAH) layers, coating on hollow mesoporous silica (HMS) spheres (step a), and pH-controlled storage (steps b, c, d) and release of drug molecules (step e) (Zhu and Li, 2005)**.

To improve living cells and delivery of bioactive molecules into the food, encapsulation is a useful tool to entrap active ingredients within carrier material. Encapsulation is an important approach to meet all demands by delivering bioactive food components at the right time and right place. Majority of materials in food encapsulation include biomolecules. Polysaccharides commonly encapsulated in food applications along with lipids and proteins. Carbohydrates also form hydrogen bond with aromas due to the presence of hydroxyl groups. Retention capacity influenced by functionality of aroma molecules (Goubet et al., 1998) and seems to decrease with polarity. Both aroma load and release kinetics comes out as unique features of sol-gel materials so, should consider and more focused during encapsulation. Encapsulation in food provide stabilization, increase in bioavailability acts as barrier between bioactive materials and environment, allowing differentiation in taste and aroma with less degradation and evaporation, making astringency of polyphenols and bitter taste, preventing chemical reaction among food components etc. it is suitable for the formation of food particles less than 40 μm size. Hydrocolloid gel-particles due to their soft solid texture, biocompatibility and perception as natural material with particle size in microns and sub-microns largely used in food, agriculture, chemical and pharmaceutical industries, as texturizer in confectionery, nutrients, slow release encapsulation of flavors and cosmetics (Burey et al., 2008). These particles form thorough linking of cross polymer chains to form three dimensional networks. Physical arrangement of junction zone is affected by different factors like temperature, inherent structure of hydrocolloid, and presence of ions. Size of colloid with their medium put effect on delectability such as sugar particles in chocolate, margarine with 22 μm in particle size and 55 μm particle size of ice-cream detected by mouth. These particles can be prepared by emulsion, extrusion, coacervation, and dispersed phase formation, ultrasonic, spray drying, and rotating disks largely used for encapsulation in dry form or in hydrated media (Figure 11). Alginate gel particles used to encapsulate vitamin C (Desai et al., 2005) and hydrocolloid particles for flavors encapsulation(Malone and Appelqvist, 2003).

High volatility of orange peel essence oil limits its practical applications. To reduce volatility and expand applications, orange peel essence oil microcapsule with β-dextrin. Method work best under conditions of encapsulation temperature 47°C (Fang et al., 2012), time 1.7 h adjusting the core/wall ratio, encapsulating rate and encapsulating yield reach upto 66.7 and 88.5% (Fang et al., 2012).

**Figure 12:**
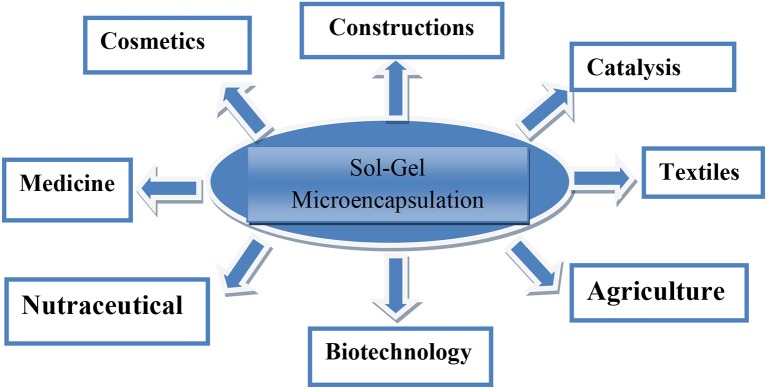
**Shows applications of sol-gel micro particles**.

**Figure 13:**
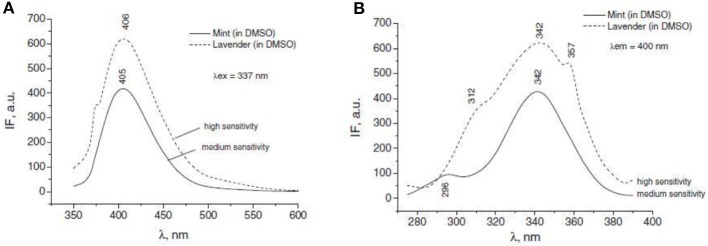
**The fluorescence emission (A) and excitation (B) spectra of the mint and lavender oils in DMSO**.

In fermentation and metabolic production processes and for the immobilization of cells and enzymes, encapsulation may be employed (Figure 12). For food encapsulation additives considered more specific, should meet safety standards of agencies such as Food and Drug administration (FDA) in USA and European food safety authority (EFSA). Functionality of encapsulation material should provide potential restrictions for coating material; type of release, stability, concentration of encapsulates, and cost constraints to the final product. Different encapsulation technologies are followed in food industry such as spray chilling, spray drying, freeze drying, molecular inclusion, melt injection, and melt extrusion (Figure 13).

**Figure 14:**
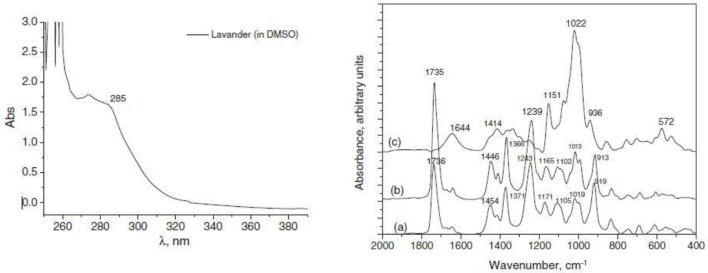
**The UV–vis absorption spectrum of lavender oil in DMSO Figure**. The IR spectra of LEO (A), inclusion complex between β-CD and LEO (B), and β-D(C) (Rǎileanu et al., 2013)

Release of aromas and fragrances from flavors encapsulated in polysaccharide gel matrix can also be studied by electronic nose methodology. It consists of array of non-specific gas sensors coupled to a pattern which allow differentiating odors. Technique applied for real time quality control and is quite simple. Researchers make a series of the study detecting changes in odor along time exploring the potential and limitations of sensor devices. Electronic nose due to high sensitivity give information about a very small change in odor, allow highly satisfactory classification of sample according to day of fragrance release of an encapsulated essence. Using raw data and supervised methods of analysis, discrimation among flavors clearly improved (Figure 14). Release of tutti frutti essence from pectin gels accounted by intensity factor (Rodriguez et al., 2010).

**Figure 15:**
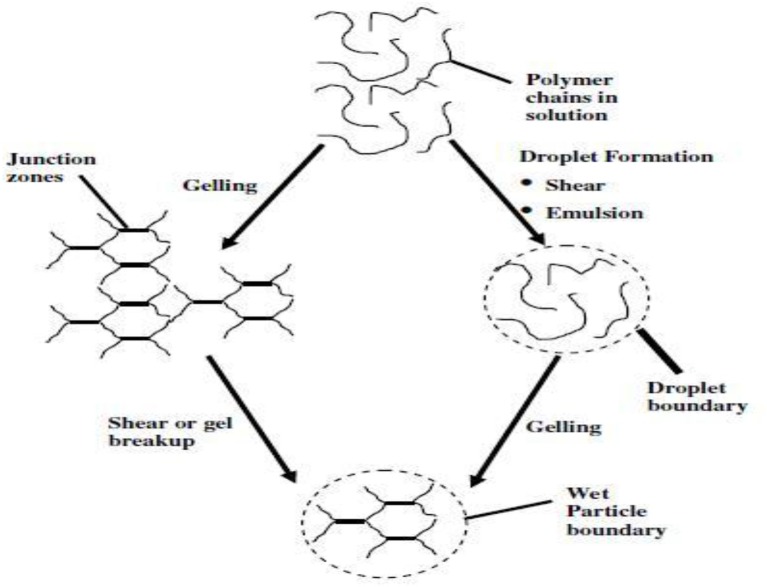
**Schematic of gel particle formation mechanisms (Burey et al., 2008)**.

**Figure 16:**
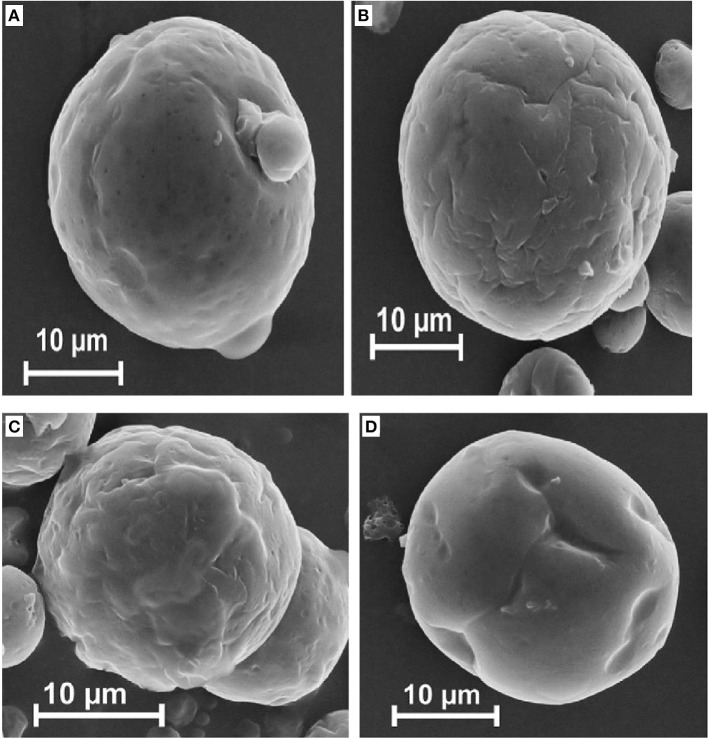
**Scanning electron micrographs of fish oil microencapsulated with different amounts of sugar beet pectin (A,D: 1.1%; B,C: 2.2%) and different oil loads (A,C: 50%; B,D: 20%) (Drusch, 2007)**.

**Figure 17:**
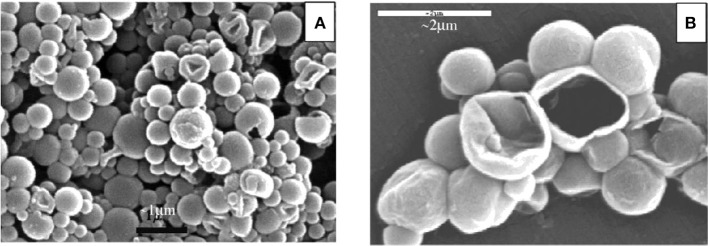
**Represents SEM micrographs of OMC containing microcapsules (A) and a rare open microcapsule (B) (Lapidot et al., 2003)**.

Sol-gel microencapsulation of fish oil rich in unsaturated fatty acids in a matrix of sugar beet pectin and glucose syrup produce an alternative encapsulating agent to milk, protein, or gum Arabics for microencapsulation of functional food ingredients with a median of less than 2 μm size and viscosity of 170 mpa S. A suitable emulsion in such case consists of 50% oil and 2.2% sugar beet pectin results in good oxidative stability. Researchers develop inexpensive and alternative polymers that could encapsulate flavors with same efficacy (Figure 15). Sugar beet pectin acts as emulsifying wall material for emulsification and it is considered as one of the key factor in spray drying. Pectin content 1–2% considered sufficient for stable feed emulsion (Figure 16). Based on physico-chemical properties of fish oil, sugar beet pectin is suitable emulsifier for lipophilic food ingredients as shown in Figure 17 (Drusch, 2007).

#### Different methods of encapsulation employed in food industry

***Extrusion Method:*** This method involves dropping of aqueous polymer solution and active agents into gelling bath. Jet cutter in comparison to other techniques found best on industrial scale applications. Coextrusion alternative to extrusion employed for forming hydrophobic and hydrophilic cores or shells (Zuidam and Eyal, 2009). For smaller particles less than 50 μm electrostatic extrusion is effective technique. ***Emulsification:*** In case of water soluble active agents, emulsification with water/oil or oil/water or water/oil/water double combinations applied. ***Fluid bed coating:*** For powder particles in batch or continuous step, encapsulation with fluid bed coating is employed. ***Spray Chilling or Spray*** ***Cooling:*** For liquid coated active agents, spray chilling, or cooling is used. Both show difference on the basis of melting point of lipids. Spray chilling range from 34 to 42°C whereas higher temperature observed for spray cooling (Gouin, 2004). ***Molecular*** ***inclusion:*** Being expensive technique utilized in cyclodextrin and liposonal vesicles. ***Vacuum and freeze drying:*** Both similar in nature possess some differences such as vacuum drying being cheaper and faster while freeze drying need high energy and long procedure time (Zuidam and Eyal, 2009). Of these commonly employed is ***spray drying*** (Nedovica et al., 2011), producing good quality particles less than 40 μm size, flexible, economic, and continuous. Technique being oldest, widely used in food industry for encapsulation of food products with sustainable flavors and fragrances with long term sustainability and stability. Advantage of this technique lie in production of flavor powder in short time. For retention and controlled release of encapsulated flavors in food many studies conducted on the influence of wall material compositions and operating conditions (Madene et al., 2006). In flavor and food industry microencapsulation of hydrophobic flavors considered most important due to good chemical stability and controlled release of solid, liquid microencapsulated food flavors. A cyclic terpene alcohol, Menthol has high volatility and whisker growth. Spray drying microencapsulation overcome these problems. In skimmed milk powder, flavors of oregano, marjoram, and citronella encapsulated by spray drying. In this technique the evaporation of solvent, that is most often water, is rapid and the entrapment of the interest compound occurs quasi-instantaneously and must be considered as an art than a science because of the many factors to optimize and the complexity of the heat and mass transfer phenomena that take place during the microcapsule formation (Gharsallaoui et al., 2007).

#### Application of sol-gel microparticles in cosmetics

**Figure 18:**
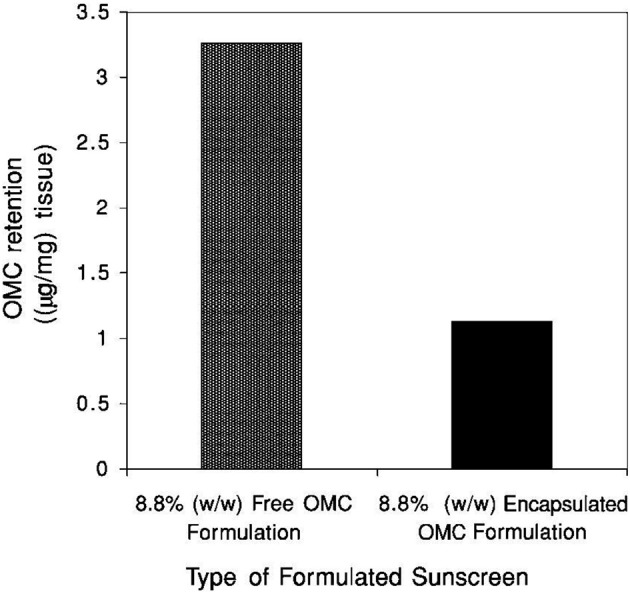
**Human epidermal penetration studies of encapsulated vs. free OMC**. The retained OMC was measured following 6 h of membrane exposure and gentle wiping of the membrane surface.

Technology find use in innovation and cosmetics as well, clearly visible to consumers avoiding side effects of organic adsorbents used in sunscreens. Molecules such as 4-methyl benzylidene camphor (4-MBC) and octylmethoxycinnamate (OMC) used initially as sunscreen but show endocrine disruption and estrogenic activity (Lapidot et al., 2003). Silica based microcapsules made from O/W emulsions ideally suited for cosmetic formulations due to clearness and smoothness. Many of cosmetics companies nowadays use organic UV filters entrapped in 1 μm transparent silica capsules formulated in water. Germanys Merck start up producing sol-gel entrapped sunscreen in early 2000 and extended a lot to supply on increasing customer base demand (Sung et al., 2014). Merck in 2001, marketed Eusolex UV- pearls as lotion, first large scale application of sol-gel doped micro particles. Generally, it is concluded that aqueous micro emulsion dispersions arouse as new opportunity for cosmetics. Extensive use of sunscreen ingredients in cosmetics against environmental exposure due to ever increasing earth's temperature causing contact dermatitis, urticaria, irritation, phytotoxic and allergic reactions, sunburns, and premature skin aging. UV-radiations divided into two categories i.e., UV-A (320–400 nm) and UV-B (290–320 nm), both damages DNA and suppresses immune system causing skin cancer. Cosmetic industry pressurized to produce safety standard products enhancing public safety. To overcome, control and eliminate these problems, sol-gel microencapsulation technology provides better and safer solution providing improved products maintaining high SPF value reducing contact between human tissues and actives. It is considered a highly flexible technique that can be applied to multiple systems, providing for the next generation of sunscreens and many as yet undiscovered applications. The technology developed uniquely enables the incorporation of high levels of sunscreen active ingredients that are much safer, as they potentially reduce the side effects associated with increased and prolonged use of these chemicals. Using this technique, transparent silica glass microencapsules ranging from 0.3 to 3 microns with UV-absorber core enclosed in silica shell reduce the penetration of sunscreen providing safety profile largely incorporated in cosmetic vehicles such as shampoo, moisturizers, lipsticks etc. achieving high sun protection factor (SPF). Encapsulation technique possesses inertness, UV-transparency based on sol-gel silica glass work well as an entrapping matrix. UV-absorber constitutes 80% of core shell capsule weight. Developed sol-gel microencapsulation technology allow to control particle size giving narrow distribution pattern shown in Figure 18 whereas core shell width of about 100 nm estimated for micro particles highlighted by drying process during sample preparation also shown in Figure 17B.

Smaller particle size gives pleasant, non gritty feel, bigger enough not to penetrate the epidermis. SPF calculations carried out by following Equation (7).

(7) $$\text{SPF }=\;\frac{\text{MED Test Material or Standard}}{\text{MED Unprotected Control}}$$

The SPF calculations obtained for each subject and represented in Table 1. SPF value of 19 signifies evenly distribution of microencapsules on the skin. By applying a physiological dose of sunscreen formulation, penetration into the dermatitis obtained. After 6 h of exposure quantity of extracted octyl methoxycinnamate (OMC) with acetonitrile described in Figure 5 showing greater penetration of non-encapsulated sunscreen than silica based sol-gel encapsulated (Lapidot et al., 2003).

**Table 1:** **SPF evaluation of a sunscreen cosmetic formulation based on the sol-gel microencapsulated sunscreens (Lapidot et al., 2003)**.

**Subject^a^**	**Skin type^b^**	**Age/Sex**	**COLIPA**	**SPF of the**
			**high standard^c^**	**formulation^d^**
AA	III	58/F	15.0	18.8
CG	II	43/F	14.8	18.5
NW	II	56/F	14.8	18.5
FC	III	34/M	15.0	15.0
TM	II	31/F	18.7	23.4
KB	II	56/F	18.7	18.7
KS	I	59/F	15.0	18.7
SB	II	45/F	18.8	18.8
JF	I	52/F	14.8	18.5
KB	III	27/M	18.7	18.7

a *Volunteers code name*.

b *Skin type category: I = Always burns easily, never tans (sensitive), II = Always burns easily; tans minimally (sensitive), and III = Burns moderatelly; tans gradually (normal)*.

c *Standard sunscreen preparation containing both UVA and UVB filters*.

d *Sunscreen formulation containing a combination of several encapsulated sunscreens*.

Oils also commonly used in perfumes, cosmetics, agriculture, and food due to aromatic properties. On the basis of origin and composition, properties of EOs can be changed by encapsulation using widely used technique called coacervation. Lemon, thyme, citronella, vanilla, menthol, eucalyptol, clove, peppermint are some of EOs used (Martins et al., 2014). In such case main purpose of encapsulation is the entrapment of core matrix into protective shell in terms of moisture resistance, controlled release, and solubility. Controlled release technique mainly adopted to deliver compounds such as drugs, fragrance and flavors, and pesticides with improved safety and efficacy (Romero Cano and Vincent, 2002).

**Figure 19:**
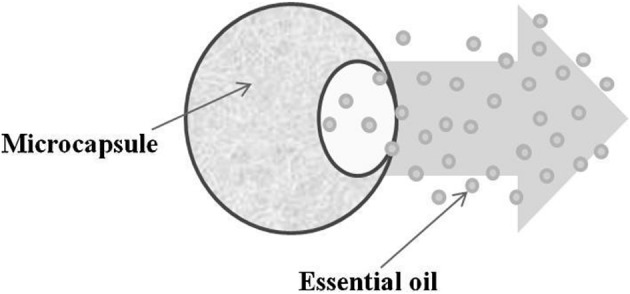
**Schematic representation of oil release through the polymeric microcapsule shell**.

Microscopy, a powerful tool used to analyze microcapsule morphology. For example: Thyme oil droplets encapsulated as spherical particles demonstrated by optical microscopy and cryogenic scanning electron microscopy confirms rough surface with some pinholes, pores, and cracks of microcapsules. Release of Thyme oil affected by film thickness and polymer concentration whereas diffusion not only affected by polymer membrane but also by type of oil used. Such difference occurs due to hydrophilic characteristic of the oil (Ashraf et al., 2013). In coacervation polar nature of the oil promotes entrapment in aqueous phase and slows down its diffusion as well (Passino et al., 2004). Another application of silica based encapsulated products found in peppermint oil in toothpaste and in skin cares proves better than non-encapsulated products. Personal care and perfumes involved three types of safe silica based encapsulation (i) Alcohol free perfumes in water to avoid skin irritation, (ii) By replacing toxic synthetic musk and stabilizing natural fragrances, (iii) Formation of perfumes of tunable scents and emergence of TOES silica based colloids also significantly drops the price of these products (Figure 19).

### Impacts of silica based materials on human health, economy and environment

Sol-gel encapsulation of silica based micro particles considered economically cheap as capital investment in manufacturing is very low and environmentally friendly. Stobber process (Stober et al., 1968) support this phenomenon by means of TEOS hydrolysis and silicic acid condensation in alcohol at room temperature.

(8) $$\begin{array}{ccc} \text{TEOS}+\text{Alcohol}+\text{Water}+\text{Catalyst}+\text{Surfactant} & \to & \text{Silica} \end{array}$$

**Figure 20:**
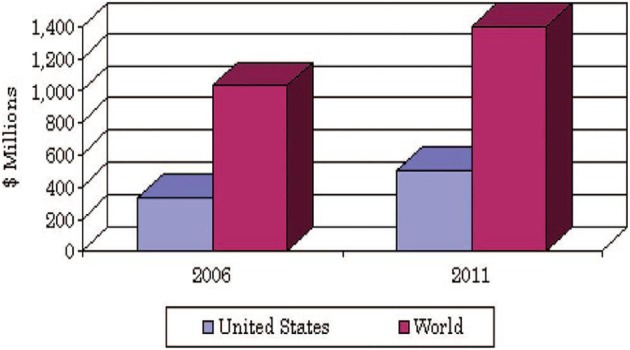
**U.S. and world markets for sol-gel products in 2006 were forecasted to increase to $1.4 billion by 2011**.

TEOS used as a cheap solvent therefore main cost of sol-gel encapsulation supposed only for labor. An overall value of sol-gel products in global market in 2006 estimated $1 billion with annual growth rate of 6.3% until 2011 as shown in Figure 20.

Positive impacts of silica based materials involving sol-gel encapsulation leading to sustainable fragrances on human health and environment apart from technical and economical feasibility are highly appreciated. Comparing synthetic perfumes which are highly poisonous causing neuromodulations in human neuroblastoma cells at extremely low concentration with EOs, being natural renewable shows multiple human health benefits. Double encapsulation of EOs first caged in β-cyclodextrin following silica encapsulation using water based route turning oil to more stabilized and resistant toward temperature, humidity, and light. Today commercialized water based products also present in tone scale in broad variety are available. In US 50 largest companies generate about 70% of revenue from this industry. This technology also enables to produce and stabilize costly active ingredients by enhancing precision and effectiveness of actions and delivered where needed in required amount. First leading Europe's chemical engineering company, sol-gel technology Ltd. constructed in 2001 installed a plant in Israel. Green technology since, 1990s grow with advancement in nanomaterial synthesis (Dahl et al., 2007). Current sol-gel encapsulation technology based on silica introduced nanobased materials into atmosphere, biosphere, and hydrosphere. Silica microspheres differ in surface chemistry with silica xerogel so it can be argued that emergence of sol-gel microspheres signifies an eminent class of sustainability.

## Conclusion

In this article, we have discussed and demonstrated encapsulation technique based on silica micro particles and its applications in different fields which offer improved products for the benefit of human beings and are environmentally friendly. Sol-gel encapsulation proves highly flexible technology that can be applied to multiple systems. Further innovation to this unique process may include isolation of incompatible compounds through their independent encapsulation within a common formulation. In order to check biological properties of encapsulated materials in food, agriculture and cosmetic industry, supplementary studies are still required.

### Conflict of interest statement

The authors declare that the research was conducted in the absence of any commercial or financial relationships that could be construed as a potential conflict of interest.
